# Beyond generic principles: a framework for the ethical analysis of artificial intelligence in organ transplantation

**DOI:** 10.3389/ti.2026.16525

**Published:** 2026-09-04

**Authors:** Nadia Primc

**Affiliations:** 1 Institute for the History and Ethics of Medicine, Medical Faculty Heidelberg, University of Heidelberg, Heidelberg, Germany; 2 Department of Biomedical Ethics and Public Health Ethics, Faculty of Health Sciences, Karl Landsteiner University, Krems, Austria

**Keywords:** artificial intelligence, distributive justice, ethics, explainability, fairness

## Abstract

Artificial intelligence (AI) is increasingly entering clinical medicine, and organ transplantation is no exception. While a growing body of literature addresses the ethical challenges of AI in healthcare, systematic analysis of the specific ethical dimensions of AI use in organ transplantation is missing. This gap reflects a genuine analytical challenge: the transplantation process is ethically complex in ways that general AI ethics frameworks do not adequately capture. Transplantation medicine simultaneously bears ethical obligations to deceased donors, living donors, donor families, organ recipients, and the public at large. These obligations shift substantially across the stages of the transplantation process, from the patient-centred ethics of donor identification and listing decisions, through the distributive justice logic governing organ allocation, to the professional responsibility questions of the peri- and post-transplant phases. A rigorous ethical analysis of AI in organ transplantation therefore requires, first, a systematic mapping of AI use cases and models onto the transplantation process; second, an identification of the stakeholder-specific ethical obligations pertinent at each stage; and third, an in-depth analysis of how those obligations are specifically affected by the properties of the AI system in question. This paper proposes this framework and illustrates it using the example of AI-assisted organ allocation.

## Introduction: a literature gap that should not exist

Artificial intelligence (AI) is transforming medicine. Organ transplantation is one of the fields in which a wide variety of use cases for the application of AI are currently being developed and discussed, from the identification of potential donors in intensive care units to AI-assisted clinical decision support for listing decisions, the planning of living donation and the prediction of graft survival. Current reviews of AI in liver transplantation identify at least ten distinct application domains, spanning pre-transplant assessment, donor-recipient matching, allocation, surgical planning, graft quality assessment, and post-transplant care [1, 2]. More recently, large language models (LLMs) have emerged as an additional category of AI with potential applications in transplantation, including administrative support, patient communication, and research assistance [3, 4].

Given this pace of development, one might expect a substantial and growing literature on the ethical challenges posed by AI in organ transplantation. A scoping review on AI in transplant surgery covering seven databases identified only 16 studies that addressed the ethical implications of AI in transplant surgery from an initial pool of 6,824 records [5]. While technical and clinical publications on AI in transplantation are proliferating, focused analysis of the ethical aspects specific to AI in organ transplantation remains largely absent. Only few empirical or normative studies place them at the centre of their investigation [5–11].

This is not a trivial gap. There is a vast amount of literature on ethical aspects of AI in healthcare and medicine. But organ transplantation is not simply another domain of clinical medicine in which general ethical principles governing AI can be straightforwardly applied without further ado to cover all the ethically relevant aspects. The transplantation process is ethically distinctive in ways that have profound implications for how AI interventions should be evaluated. The purpose of this paper is to articulate why AI in organ transplantation calls for an ethical analysis of its own, to propose a methodological framework for developing such an ethical analysis, and to illustrate how this framework can guide ethical analysis of AI in organ transplantation.

## Why organ transplantation poses distinctive ethical challenges

Organ transplantation occupies a unique position in the landscape of clinical medicine. Several features of transplantation create specific ethical obligations and tensions that are absent or far less acute in other fields of patient care [12–14].

Most important, organ transplantation largely depends on a scarce, non-reproducible, public resource. Organs become available through the consent of donors or their families. Every allocation decision is simultaneously a clinical decision that mainly takes place between a patient and the healthcare team and a distributive justice decision that transcends this individual relationship: every organ given to one patient is an organ unavailable to another. The ethical framework of distributive justice stands in tension with the patient-centred logic that guides most clinical decision-making, where the healthcare team is expected to act in the best interest of the individual patient [13]. Distributive justice, by contrast, asks how a scarce resource can be fairly distributed among the competing claims of different patients. What is in the best interest of one individual patient may not align with what constitutes a fair distribution among all patients awaiting transplantation. Organ allocation is a decision that transcends the individual clinical relationship. The public has a legitimate stake in how this common resource is managed, which is why allocation of organs is generally governed by nation-wide allocation guidelines [13].

Furthermore, the transplantation process involves a uniquely diverse set of stakeholders, each bearing distinct and sometimes competing ethical claims. Potential deceased donors have expressed or presumed wishes that must be respected. Living donors accept medical risk for the benefit of others, which represents a morally exceptional act that generates ethical obligations on the part of transplant teams. Waitlisted patients have rights to equitable access to a life-saving therapy, but on the other side are also confronted with public demands regarding the responsible use of the scarce resource of organs [15]. And society has an interest in a system that is transparent, fair, and worthy of public trust [16].

The ethical landscape shifts substantially across the stages of the transplantation process. At the stage of donor identification and listing decisions, central ethical issues concern the rights of patients, as well as their families [17]. These ethical claims primarily concern the individual relationship between the healthcare team and patients or their families, but also touch ethical obligations of institutions such as hospitals or the healthcare system as a whole. However, the fundamental orientation of the ethical considerations in this part of the transplantation process can be described as patient-centred.

At the stage of organ allocation, the ethical focus shifts toward distributive justice and an allocation-centred logic: how should competing claims be weighed, and by what criteria? This encompasses a fundamental tension between several allocation principles, notably urgency (prioritising the sickest patients) and utility (post-transplant survival), as well as considerations of fairness. The latter includes not only the question of how a fair balancing of these two principles might look like – to which consequentialist (i.e., outcome-oriented) and deontological (e.g., rights- and intentions-oriented) ethics give differing answers. But also, how further concerns for fairness should be integrated into the allocation scheme, e.g., the prioritization of paediatric or highly sensitized candidates, geographic inequity, or gender-, ethnicity- and socio-economic-related inequities [18].

During the surgical and perioperative phases, the ethical focus shifts again, back to a more patient-centred ethical stance, although with more stakeholders involved than at the first stages of the transplantation process. Questions of professional responsibility within the patient-doctor-relationship and the interprofessional team come to the fore. In the long-term follow-up of recipients and living donors, issues of adherence and responsibility for graft survival, monitoring of immunosuppression and associated risks, equity in access to specialised care, become central.

The past decade has seen the emergence of a substantial body of ethical reflection on AI in medicine, crystallised in national and international guidelines, professional position statements, and academic literature. Core ethical requirements, e.g., transparency and explainability, bias and discrimination, privacy and data protection, accountability, the preservation of human oversight, are broadly agreed upon and have been applied to the transplantation context in recent reviews [2, 5, 8, 12, 19]. These requirements are not irrelevant to organ transplantation, but they are rather generic. Most publications addressing AI in transplantation focus on clinical efficiency and outcomes, with only superficial treatment of ethical dimensions [5]. This need has been recognised at the level of professional societies: the recent ESOT position paper on AI in transplantation explicitly identifies the move from generic ethical principles to transplantation-specific ethical guidance as a critical priority [12]. The present paper addresses this challenge by proposing a methodological framework for how such transplantation-specific analysis can be structured. Moving from principles to practice in the ethics of AI in transplantation requires a more structured and context-sensitive framework by taking into account the above-mentioned stage-specific considerations, as ethical analyses will otherwise inevitably miss important ethical dimensions of the problem.

## A framework for analysing ethical aspects of AI in organ transplantation: mapping use cases, process stages, and ethical stakes

The development of a rigorous ethical framework that could guide future research on ethical aspects of AI in organ transplantation requires three integrated analytical steps.

### Step 1: systematic mapping of AI use cases and their respective AI models onto the transplantation process

As a prerequisite for a comprehensive ethical framework for AI in organ transplantation, a systematic overview of existing and emerging AI use cases across the transplantation process is required. A crucial analytical distinction must be established at the outset: between an AI use case, i.e., the clinical task being addressed, defined by the question “What problem are we solving, and where in the transplantation process does it occur?”, and the specific AI model or system deployed to address it, defined by the question “What technology is doing the work, and how?” This distinction is not merely technical, it has direct ethical significance, for two reasons.

First, the same use case may be addressed by various AI models with very different ethical profiles. Consider clinical decision support for patients facing the choice between dialysis and kidney transplantation. This use case can be addressed by a classical statistical prediction model, as illustrated by “iChoose Kidney”, which uses logistic regression to provide individualised mortality risk estimates comparing dialysis versus transplantation, designed as a shared decision-making tool for clinicians and patients [2]. The same use case could in principle be addressed by a more complex ML model. Depending on the architecture of such a model, this substitution could allow to integrate more factors into the predictive model, but simultaneously introduce additional ethical challenges: more opaque models are harder to explain to patients and clinicians, potentially undermining the informed and shared character of the decision-making process that this use case is specifically designed to support, or making it more difficult to identify ethically questionable criterions used in the model to make that prediction [10].

Second, the same model type may serve use cases with fundamentally different ethical stakes. A predictive ML model used to support shared decision-making in the clinical consultation operates within a patient-centred ethical framework, where the primary obligations concern individual autonomy, informed consent, and the quality of the clinician–patient relationship. The same class of predictive ML model, deployed for organ allocation, operates within an entirely different ethical register, that is governed by distributive justice, equity, and the competing claims of multiple patients to a scarce public resource.

This first step of the mapping exercise should also record the current stage of development and clinical implementation of each AI system identified. While digitalised classical statistical prediction models (such as “iChoose Kidney”) have reached clinical deployment, ML-based approaches in the stricter sense remain largely at the research, development, or pilot stage [1, 2, 5, 8, 18]. This lack of clinical implementation is itself an important result of a systematic ethical analysis as it suggests that the governance frameworks, validation standards, and accountability structures required for the responsible use of these more complex systems have, in most cases, not yet been established.

### Step 2: identifying stakeholder-specific ethical obligations across the transplantation process

Where Step 1 is primarily descriptive-empirical, Step 2 is normative in character. Its task is to identify, for each stage of the transplantation process, the relevant stakeholder groups and the ethical demands and obligations attributed to each. As argued above, the ethical landscape of transplantation is not uniform: it shifts substantially across the stages of the process. These shifts matter because they determine which ethical framework and ethical issues are primarily at stake when an AI system is introduced at a given point in the process. This step requires specifying, for each process stage, the relevant stakeholder groups, the nature of the ethical demands and obligations relevant to each, and whether those are primarily governed by a patient-centred, a justice-oriented ethical logic, or even both. The output of Step 2 is therefore a normative map of the transplantation process: a structured account of who bears what obligations toward whom, and within what ethical framework, at each stage.

### Step 3: in-depth ethical analysis of specific use cases

The first two steps create the conditions for the third and integrative step: targeted, in-depth and comparative ethical analyses of specific AI use cases and their models. By combining the descriptive-empirical output of Step 1 with the normative output of Step 2 it becomes possible to ask precisely the right ethical questions about a specific system. What are the ethically relevant properties of this particular use case and model, given the ethical demands, obligations and stakeholder relationships characteristic of the stage at which it operates? This third step is genuinely integrative: it brings the empirical specificity of the AI system into direct engagement with the normative structure of the transplantation context.

**FIGURE 1:**
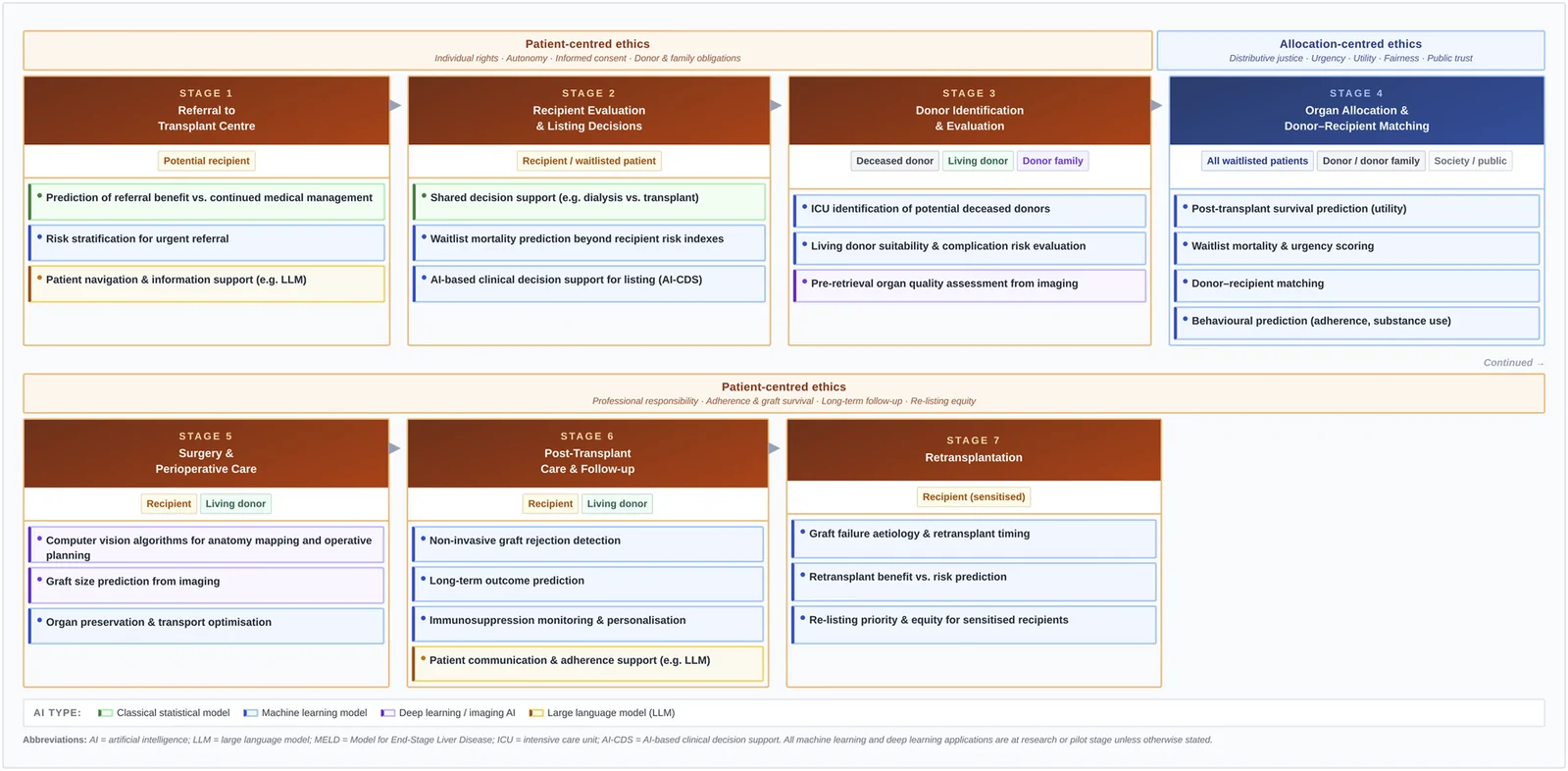
Structured process diagram arranged in two rows illustrating seven sequential stages of organ transplantation onto which AI use cases are mapped. Stages 1 to 3 and 5 to 7 are framed under patient-centred ethics; Stage 4, organ allocation and donor-recipient matching, is framed under allocation-centred ethics governed by distributive justice. Each stage column displays stakeholder badges identifying the parties whose interests are ethically at stake at that stage, and coded cards representing individual AI use cases. Cards are categorised by AI type: classical statistical model, machine learning model, deep learning and imaging AI, or large language model. A legend and abbreviations list appear at the bottom.

Figure 1 provides a tentative illustration of how such a mapping of AI use cases and models onto the transplantation process might be structured.

## Illustrating the framework: AI in organ allocation

Given the constraints of this point of view paper, the following can only outline how a full three-step analysis would look when applied to the use case of AI-assisted organ allocation. The execution and presentation of the ethical analysis must be the subject of future research, such as that being carried out by the newly established ELPAT task force on AI in organ transplantation. AI-assisted organ allocation is chosen as an example here as it is an extensively discussed use cases in the emerging literature, and one that sits at the intersection of the most acute ethical tensions in transplantation medicine.

As a first step of an ethical analysis, one would need to systematically map the AI use cases and models deployed at the allocation stage of the transplantation process. A brief survey of the current literature reveals a cluster of applications: waitlist mortality prediction, post-transplant graft and patient survival prediction, and donor-recipient matching, addressed by a range of ML models. Systematic reviews consistently show that the latter outperform classical scoring systems such as the Model of Endstage Liver Disease (MELD), Kidney Donor Risk Index (KDRI), or Estimated Post-Transplant Survival (EPTS) in predictive accuracy, but that their translation into ethically governed allocation systems remains substantially incomplete [18–20]. This mapping step also requires recording an ethically relevant property of each model type, especially the trade-off between predictive accuracy and explainabilty [10]. Ensemble methods such as gradient boosting offer reasonable transparency through feature importance scores, while deep learning architectures tend to achieve higher predictive accuracy at the cost of opacity, making it substantially harder to explain why a given patient was ranked as they were [12, 18].

As a second step, one would need to identify the stakeholder-specific ethical obligations characteristic of the allocation stage. Organ allocation is governed by an ethical logic fundamentally different from the patient-centred frameworks dominant at other stages of the transplantation process. This means that the prediction models used in organ allocation raises different ethical questions than similar models that are used in the more patient-centred decision to accept an organ offer or not. As mentioned above, the primary ethical obligations of transplant professionals at this stage are therefore distributive justice, fairness, transparency, and the maintenance of public trust, not the autonomy and individualised care that dominate at other stages of the process.

As a third step, one would bring these obligations into direct engagement with the specific ethical properties of the ML systems identified in Step 1. For example, it may be more acceptable to use an opaque but more accurate prediction model in the patient-centred decision to accept an organ offer (provided the patient consents to its use), than at the stage of organ allocation, which, for reasons of distributive justice, requires a greater degree of explainability even at the cost of lower accuracy [10]. Also, explainability tools may illuminate individual predictions but do not constitute allocation-level auditability, such that the prioritisation logic governing which patients are ranked above others may remain opaque even where individual predictions are interpretable. Conversely, the ethical acceptability of the criteria used by a model may itself vary by stage. For example, the integration of behavioural predictions, such as medication adherence or alcohol use, may be judged differently depending on whether it is used at the allocation or individual level. Furthermore, ML models trained on historical transplant registry data are liable to encode and amplify existing health inequities known to be relevant in transplant medicine (e.g., disparities in access attributable to socioeconomic, geographic, or ethnic factors embedded in the data but not reducible to clinical variables).

None of these concerns are addressable by the application of generic AI ethics principles alone. Each requires the kind of integrated analysis the framework proposes: grounded in the specific distributive justice logic of the allocation stage, attentive to the stakeholder relationships and obligations characteristic of that stage, and directed at the concrete ethical properties of the ML systems under development. This illustrates the broader argument presented in this article: the ethical challenges of AI in organ transplantation are not a subset of the ethical challenges of AI in medicine generally, but a domain requiring analysis of its own.

## Conclusion: towards an interdisciplinary ethical analysis of AI in organ transplantation

The ethics of AI in organ transplantation is an emerging field that has not yet received the systematic scholarly and policy attention it warrants. As AI applications in transplantation move from the research pipeline into clinical practice, the urgency of closing this gap is increasing. The framework proposed here offers a methodologically rigorous approach to the task. It builds on established methods in transplantation ethics and adapts them to the specific challenges of algorithmic decision-making.

This work is inherently interdisciplinary. A thorough ethical analysis of AI in organ transplantation requires sustained collaboration across transplant medicine, bioethics, law, the social sciences, psychology, AI research, as well as patient representatives. No single discipline possesses the knowledge base required to conduct this analysis adequately.

The ELPAT task force on AI in organ transplantation, has taken up this agenda. The ELPAT task force formally commenced its work in late 2025 and is currently engaged in the first phase of use case mapping. Individuals and research groups who wish to contribute to this interdisciplinary effort are warmly invited to contact the author. The ethical challenges of AI in organ transplantation are too important and too specific to be left to general frameworks alone [21].

## Data Availability

The original contributions presented in the study are included in the article/supplementary material, further inquiries can be directed to the corresponding author.
